# Light regulates the synthesis and accumulation of plant secondary metabolites

**DOI:** 10.3389/fpls.2025.1644472

**Published:** 2025-08-04

**Authors:** Wenyuan Wu, Huan Wu, Rentao Liang, Shiping Huang, Luxiao Meng, Miao Zhang, Fengfeng Xie, Hua Zhu

**Affiliations:** ^1^ Guangxi Key Laboratory of Zhuang and Yao Ethnic Medicine, Guangxi University of Chinese Medicine, Nanning, China; ^2^ The Development and Industrialization of Zhuang and Yao Ethnic Medicinal Materials of Guangxi Universities Engineering Research Center, Guangxi University of Chinese Medicine, Nanning, China; ^3^ The Collaborative Innovation Center of Zhuang and Yao Ethnic Medicine, Guangxi University of Chinese Medicine, Nanning, China; ^4^ Guangxi Engineering Research Center of Ethnic Medicine Resources and Application, Guangxi University of Chinese Medicine, Nanning, China

**Keywords:** light, plant, secondary metabolites, light quality, light intensity, photoperiod

## Abstract

Secondary metabolites are low-molecular-weight organic compounds produced by plants under specific conditions. While they are not directly involved in fundamental growth and developmental processes, they play crucial roles in plant defense, protection, and regulation. These compounds mainly include phenolics, terpenoids, alkaloids, flavonoids, and others. Light, as a key environmental factor regulating the synthesis of plant secondary metabolites, influences their production and accumulation through multidimensional regulatory mechanisms. Different light qualities activate or suppress specific metabolic pathways via signal transduction networks mediated by specialized photoreceptors. Light intensity dynamically modulates secondary metabolite accumulation by affecting photosynthetic efficiency, while photoperiod coordinates metabolic rhythms through circadian clock genes. These light responsive mechanisms constitute a chemical defense strategy that enables plants to adapt to their environment, while also providing critical targets for the directed regulation of medicinal components and functional nutrients. This study provides a review of recent research on the effects of light on plant secondary metabolites, aiming to deepen the understanding of the molecular mechanisms underlying light-regulated secondary metabolism. The findings may offer an insight for enhancing bioactive compounds in medicinal plants and developing functional agricultural products.

## 1 Introduction

During the long evolutionary process, plants have developed a series of secondary metabolites with unique physiological functions, primarily including flavonoid (Ma et al., 2024), terpenoid (Pichersky and Raguso, 2018), alkaloid (Gu et al., 2025) and phenolic (Sheteiwy et al., 2023) compounds. These specialized metabolites not only help plants cope with environmental stresses but also play pivotal roles in fields such as medicine and health, nutritional food, and agricultural production (Brosset and Blande, 2022; Ahmed et al., 2024; Jahan et al., 2025). Among various environmental factors affecting plant growth, the light environment - with its unique spatial distribution, spectral properties, irradiation intensity, photoperiod, and circadian rhythms - elicits distinct physiological responses in plants and profoundly influences the biosynthesis and accumulation of secondary metabolites (Morales et al., 2025). Breakthroughs in modern photobiological research and metabolomics analysis techniques have increasingly elucidated the molecular mechanisms by which light signals regulate plant secondary metabolism, paving innovative pathways for using optical regulation technologies to enhance the content of functional plant compounds.

The regulatory effects of light environment on plant secondary metabolism exhibit multi-dimensional characteristics. From the perspective of spectral properties, specific wavelengths of light can achieve differential biological regulation through specialized photoreceptor systems. UV, for instance, activates the HY5 transcription factor signaling pathway via the UVR8 receptor, enhancing the biosynthesis efficiency of phenolics, flavonoids, and anthocyanins (Shamala et al., 2020; Morales et al., 2025). Blue light, mediated by cryptochrome and phototropin protein complexes, influences the phenylpropanoid metabolism process by acting on transcriptional regulatory networks such as HY5 and MYB (Rai et al., 2019; Wang et al., 2020a; Zhang et al., 2025b). Red light, on the other hand, modulates the production of terpenoids through phytochrome mediated hormonal signaling pathways, altering the levels of endogenous hormones (Holalu et al., 2020; Escobar-Bravo et al., 2024). The interactions between these specific light wavelengths and photoreceptors elucidate the response mechanisms of photoreceptors to light signals. In terms of light intensity, it regulates the expression of metabolism-related genes by modifying photosynthetic efficiency, energy allocation, and stress responses, ultimately affecting the accumulation of metabolic products (Borbély et al., 2022; Zhu et al., 2024). However, excessive light may induce photoinhibition, disrupting normal metabolic activities (Alsharafa et al., 2014). On the temporal scale, the circadian rhythm system coordinates the synthesis of plant secondary metabolites by perceiving changes in photoperiod (Hoffman et al., 2010). Thus, Understanding the molecular regulatory mechanisms of light signals on plant secondary metabolism holds significant scientific importance and practical value. On one hand, it can reveal the molecular regulatory networks by which plants adapt to their external environment; on the other hand, it provides theoretical support for developing light-based technologies to improve crop quality (Zhang et al., 2024c). This review summarizes the effects of different light characteristics (light quality, intensity, and photoperiod) on plant secondary metabolites and their underlying mechanisms, highlights the progress in the application of light-control technologies in agriculture and medicine, and explores future research directions in this field. The aim is to provide insights for both fundamental research and industrial applications of light-mediated regulation in plant secondary metabolism.

## 2 Effects of light quality on plant secondary metabolites

**Figure 1:**
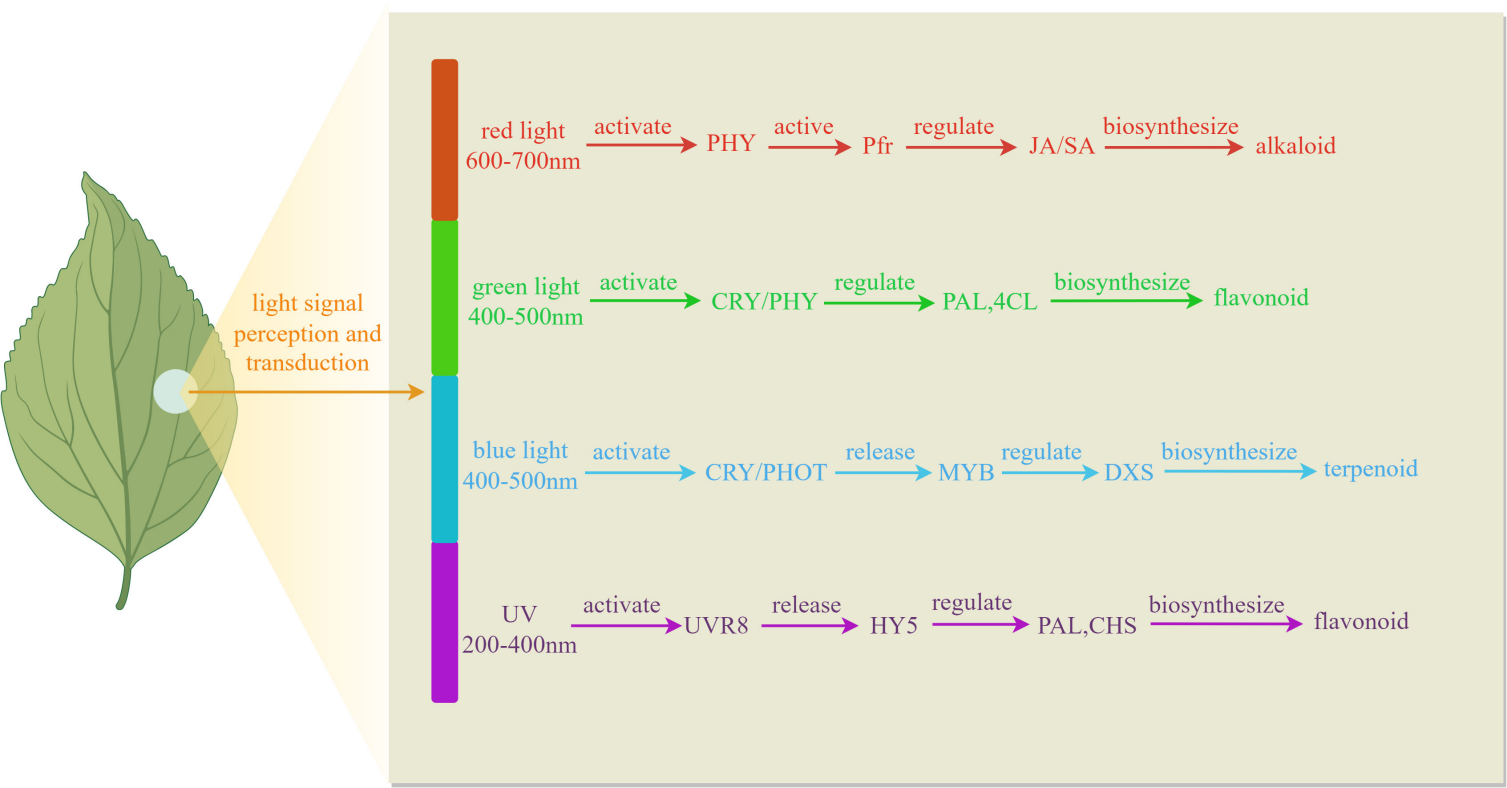
Mechanism of different light quality regulating plant secondary metabolites.

Light quality, as a crucial parameter of the light environment, exerts multidimensional regulatory effects on plant growth, development, and physiological metabolism through specific wavelength combinations. In plant physiology and facility agriculture, light sources can be categorized into visible light, infrared light, and ultraviolet light based on their wavelengths. Among these, visible light can be further divided into various colors such as red, orange, yellow, green, blue, indigo, and violet, which play a regulatory role in plant growth, development, and the accumulation of metabolic products. As illustrated in **Figure 1**, different wavelengths of light signals are selectively recognized by the plant photoreceptor system, triggering distinct physiological responses that regulate the synthesis of secondary metabolites. As shown in **Table 1**, differential light treatments influence the expression of different genes.

**Table 1:** Effect of light quality on accumulation of secondary metabolites in plants.

Species	Light quality	Treatment time	Regulated genes/proteins	Secondary metabolites	References
*Ocimum basilicum*	supplement UV-B TL, 311nm	4 days	up-regulating CHS	total flavonoid↑	(Skowron et al., 2025)
*Oryza sativa*	blue lamp	10 h d^-1^ for 9 days	up-regulating PAL, 4CL, CHS, CHI, F3H, FLS	flavonoid↑	(Zhang et al., 2025b)
*Atropa belladonna*	red LED, 620-660nm	16 h d^-1^ for 35 days	up-regulating GDHA, At2g42690, PAO5	hyoscyamine, scopolamine↑	(Gu et al., 2025)
*Scots Pine*	red/far-red LED, 660/720 nm	14 h d^-1^ for 40 days	up-regulating CHS, JAZa	proanthocyanidins, catechins↑	(Pashkovskiy et al., 2024)
*Mangifera indica*	UV-B/white LED, 312 nm	24 h d^-1^ for 14 days	up-regulating MYB, C2H2, HSF, C3H, bHLH	anthocyanins, flavonoids, phenolics↑	(Yang et al., 2024)
*Oryza sativa*	blue lamp	14 days	up-regulating PAL, 4CL, CCR, POD, CHS, CHI, FLS, ANR	JA, flavonoids↑	(Zhang et al., 2024b)
*Wheat Sprouts*	red LED, 660-665nm	18.2 h d^-1^ for 4 days	up-regulating PAL, C4H, 4CL	total phenolic↑	(Zhang et al., 2024a)
*Taxus wallichiana*	UV-B FL, 280-320nm	48 h	up-regulating Bapt, Dbtnbt;down-regulating CoA, Ts, Dbat	cephalomannine, paclitaxel↑	(Zhong et al., 2024)
*Artemisia argyi*	UV-B, 280-315nm	6 days	up-regulating HY5, bHLH25, bHLH18, bHLH148,MYB114, MYB12, MYB111	terpenoids, phenolic↑	(Gu et al., 2024)
*Sage bulleyana*	red LED, 660/730nm	18 h d^-1^ for 35 days	up-regulating PAL, TAT, RAS	total polyphenol↑	(Grzegorczyk-Karolak et al., 2025)
*Brassica oleracea*	white LED,450-660nm	16 h d^-1^ for 10 days	up-regulating BoPDS, BoZDS	carotenoid↑	(Lee et al., 2023)
*Rhododendron chrysanthum*	UV-B TL, 295nm	8 h d^-1^ for 2 days	up-regulating DHD, SDH	total phenolic ↑	(Sun et al., 2024)
*Scots Pine*	red LED, 660nm	16 h d^-1^ for 42 days	up-regulating 4CL, LAR, PR1, PR5, JAZa, JAZb, MYC	proanthocyanidins,flavonoids catechins↑	(Pashkovskiy et al., 2023)
*Musa nana*	UV-C TL	18 days	up-regulating PAL, POD, MYB, bHLH, COI1, JAR1, MYC2	total phenols, flavonoids, alkaloid↑	(Chen et al., 2022)
*Ocimum basilicum*	UV-A LED, 365-399nm	16 h d^-1^ for 14 days	up-regulating PAL	phenolic ↑	(Kang et al., 2022)
*Morus alba*	UV-B mercury vapor lamp, 280-320nm	15 mins	up-regulating PAL,CHI, LAR	proanthocyanins, moracin N, chalcomaricin↑	(Li et al., 2022b)
*Melissa officinalis*	70%R/30%B LED, 650/460nm	16 h d^-1^ for 49 days	up-regulating DAHPS, TAT, RAS	total phenolics, rosmarinic acid↑	(Ahmadi et al., 2022)
*Brassica napus*	supplement UV-B TL, 280-315nm	3 days	up-regulating PAL, C4H, 4CL, CHS, CHI, F3H, FLS, F3`H, DFR	phenylpropanoid, flavonoid, anthocyanin↑	(Lee et al., 2022)
*Cajanus cajan*	UV-B lamp, 313nm	42 days	up-regulating CHS, STS	phenolic↑	(Gai et al., 2022)
*Brassica alboglabra*	UV-A LED, 370-390nm	12 h d^-1^ for 12 days	up-regulating DOF1.1, MYB41, MYB28, MYB34, BCATs, MAMs, CYP79s, CYP83s, AOPs	glucosinolates↑	(He et al., 2021)
*Artemisia annua*	UV-B LED, 275-320nm	16 h d^-1^ for 28 days	up-regulating ADS, MYB, NAC	artemisinin↑	(Ma et al., 2020)
*Prunus persica*	blue LED, 445nm	8 days	up-regulating PpPSY; down-regulating PpCCD4, PpNCED1, PpNCED2	carotenoid↑	(Zhang et al., 2025c)
*Salvia miltiorrhiza*	UV-B lamp, 313nm	16 h	up-regulating NAC1, PAL3, TAT3	salvianolic acid↑	(Yin et al., 2020)
*Vitis amurensis*	UV-C lamp, 254 nm	10 mins	up-regulating STS	resveratrol↑	(Yin et al., 2016)
*Salvia miltiorrhiza*	blue LED, 450nm	16 h d^-1^ for 21 days	down-regulating HMGR, DXS2, DXR, GGPPS, CPS, CYP76AH1	tanshinone IIA↓	(Chen et al., 2018)

### 2.1 UV light

**Figure 2:**
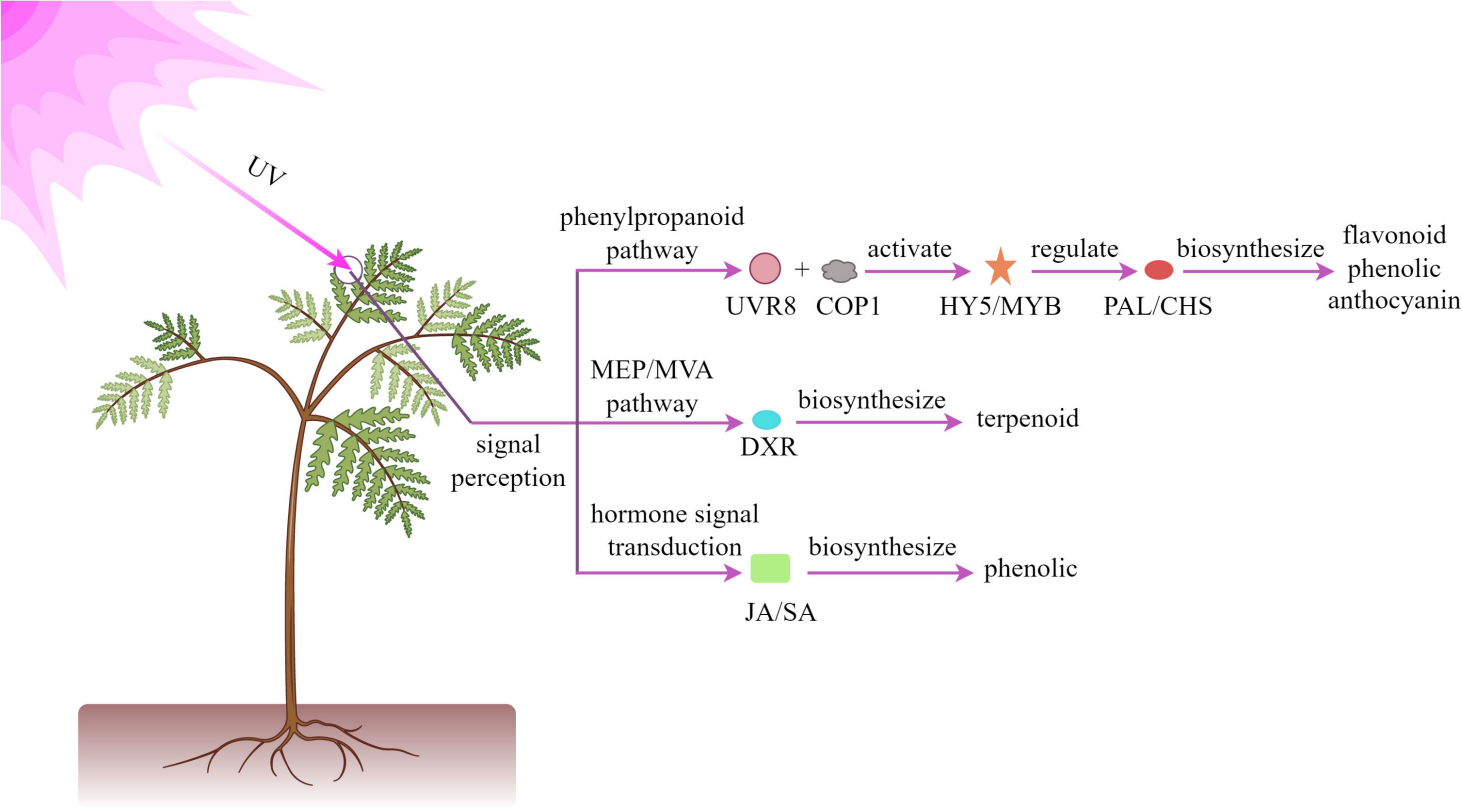
Mechanism of UV light regulating plant secondary metabolites.

UV radiation, as a significant component of the solar spectrum, can be classified into three primary bands based on wavelength range: UV-A (315–400 nm), UV-B (280–315 nm), and UV-C (200–280 nm). When plants are exposed to UV radiation, they activate a series of defense mechanisms, primarily manifested through the enhanced biosynthesis of specific secondary metabolites, including flavonoids, phenolics, and terpenoids. These substances play crucial roles in plant responses to environmental stresses (Vanhaelewyn et al., 2020; Kivimäenpä et al., 2022). As illustrated in **Figure 2**, at the molecular level, UV radiation can specifically activate photoreceptor system in plants, promoting the combination of UVR8 photoreceptors with COP1, activating HY5 transcription factor. This subsequently induces the expression of key enzymes in the phenylpropanoid pathway, such as PAL and CHS, thereby enhancing the synthesis and accumulation of anthocyanins and flavonoids. These biochemical responses significantly improve the plant’s resistance to oxidative stress (Shamala et al., 2020; Jaiswal and Agrawal, 2021). Moreover, the terpenoid biosynthetic gene network is dynamically regulated through both the MEP and MVA pathways, ultimately modulating terpenoid diversity and yield (Contreras-Avilés et al., 2024; Zha et al., 2024). Regarding signal transduction, ultraviolet radiation influences the biosynthesis of defensive compounds such as phenolic acids by regulating phytohormone JA and SA pathways (Gai et al., 2022; Sun et al., 2024; Zhou et al., 2025).

Different UV treatments can improve the nutritional quality of agricultural products and the component content of medicinal plants, while their light signals also vary in regulating the biosynthetic genes and hormonal signaling mechanisms in plants. UV-A treatment of *Ocimum basilicum* upregulates PAL enzyme activity, increasing total phenolic concentration and antioxidant capacity (Kang et al., 2022). UV-A significantly enhances the content of gallotannins and ellagitannins in *Eucalyptus camaldulensis* by altering the expression of phenolic compounds (Khanal et al., 2025). Under UV-A exposure, the shikimate and MEP pathways are modulated to promote the synthesis of flavonoids and phenolic acids in *Lactuca sativa*, while the MVA pathway is suppressed, reducing the biosynthesis of sesquiterpenes and triterpenes (Zha et al., 2024). Under UV-B exposure, the total flavonoid and phenolic content is increased in *Mangifera indica* (Yang et al., 2024), *Stevia rebaudiana* (Semenova et al., 2024), *Lactuca sativa* (Skowron et al., 2024), *Pelargonium graveolens* (Jadidi et al., 2023), *Eucommia ulmoides* (Xiao et al., 2023), and *Oryza sativa* (Aimvijarn et al., 2023), enhancing their antioxidant activity. In *Vitis vinifera*, in addition to boosting secondary metabolite levels, also improves flavor (Narra et al., 2023). UV-B acts on *Pennisetum glaucum* to increase the content of phytosterols and triterpenoids (Singh and Choudhary, 2025).

Furthermore, UV-B treatment modulates the gene expression involved in plant secondary metabolism. For instance, upon UV-B irradiation, genes such as PAL, C4H, 4CL, CHS, and CHI in *Brassica napus* are rapidly upregulated within 24 hours, promoting the accumulation of phenylpropanoids, flavonoids, and anthocyanins (Lee et al., 2022). After 48 hours of UV-B exposure in *Taxus wallichiana*, the expression of Bapt and Dbtnbt genes is upregulated, while CoA, Ts, and Dbat genes are downregulated, leading to increased synthesis of paclitaxel and cephalomannine (Zhong et al., 2024). Through the shikimate and MEP pathways, genes including HY5, bHLH25, bHLH18, bHLH148, MYB114, MYB12, and MYB111 in *Artemisia argyi* are upregulated, enhancing the production of terpenoid and phenolic compounds (Gu et al., 2024). A 15-minute UV-B irradiation followed by 36 hours of dark incubation upregulates PAL, CHI, and LAR gene expression in *Morus alba*, promoting the accumulation of proanthocyanins, moracin N, and chalcomaricin, which indicated that appropriate dark incubation under stress conditions facilitates secondary metabolite biosynthesis (Takshak and Agrawal, 2019; Li et al., 2022b). Additionally, in *Salicornia europaea*, the ABA pathway under UV-B is regulated by ROS, resulting in reduced ABA levels and increased carotenoid content, thereby improving nutritional quality (Fitzner et al., 2023). Regarding UV-C, it upregulates the expression of genes such as PAL, POD, MYB, bHLH, COI1, JAR1, and MYC2 by modulating flavonoid and phenylpropanoid biosynthetic pathways and activating phytohormones, thereby enhancing the accumulation of alkaloids, flavonoids, and phenolics in *Musa nana* (Chen et al., 2022). Plant responses to UV radiation exhibit significant species-specificity and developmental stage-dependence. Tolerance thresholds to UV radiation vary markedly across plant families and even within the same species at different growth stages (Kang et al., 2022). This characteristic holds crucial implications for modern agricultural practices, particularly in controlled-environment agriculture systems where precise light quality management must be tailored to crop varieties and growth phases. Notably, UV intensities exceeding plant tolerance thresholds can cause photosystem damage and inhibit normal growth, exemplifying the biphasic “low-dose stimulation, high-dose inhibition” response typical of UV-induced physiological effects.

### 2.2 Blue light

**Figure 3:**
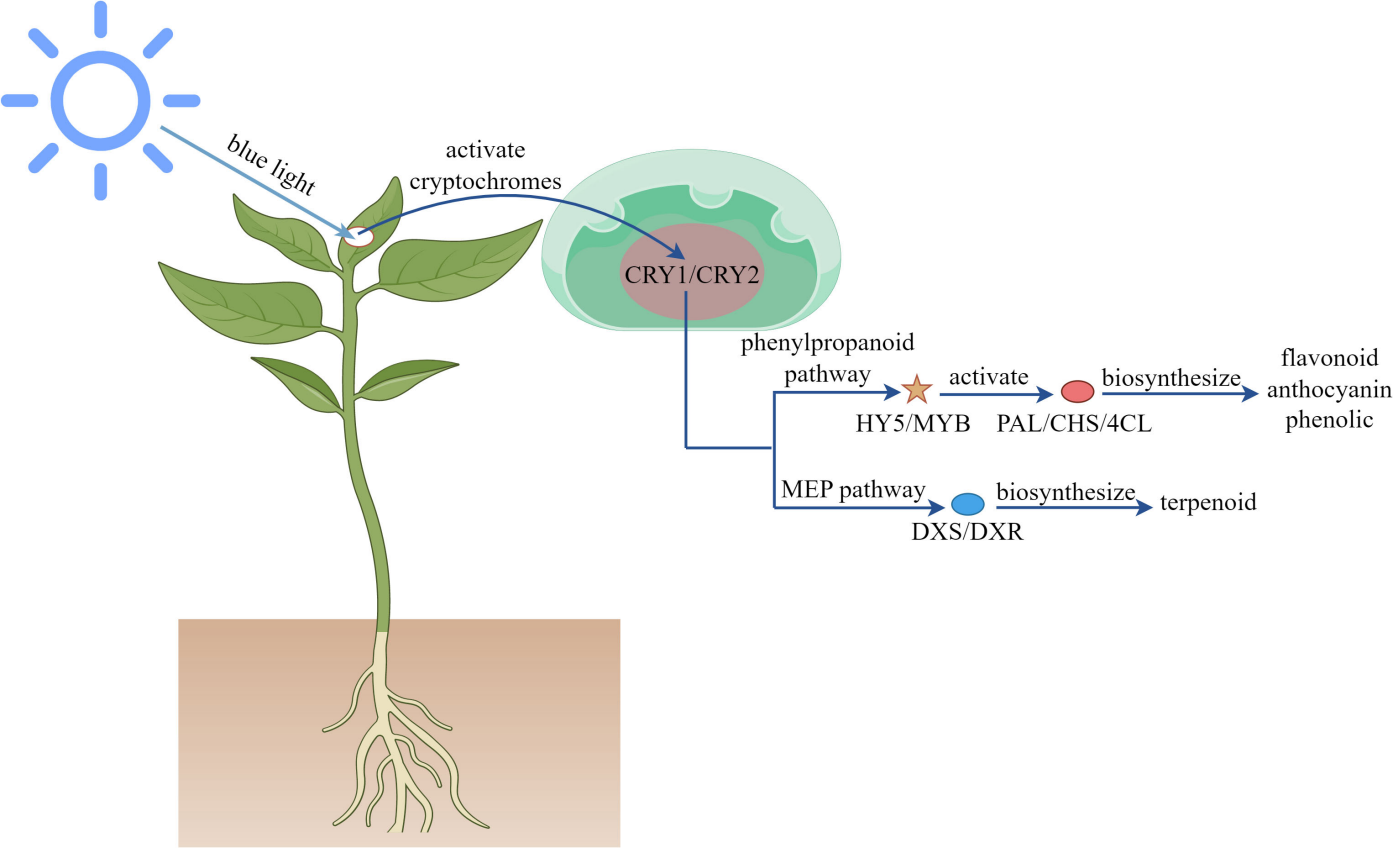
Mechanism of blue light regulating plant secondary metabolites.

As a vital component of the visible spectrum, blue light plays a crucial regulatory role in plant growth and development. As shown in **Figure 3**, this specific wavelength activates the plant photoreceptor system, including cryptochromes and phototropins, to mediate photomorphogenesis-inhibiting hypocotyl elongation while promoting leaf expansion and stomatal opening (Wang et al., 2017; Gao et al., 2022; Li et al., 2022a). At the metabolic level, blue light upregulates the expression of transcription factors such as HY5 and MYB, which specifically activated the expression of key enzymes of phenylpropanoid pathway PAL, CHS and 4CL, promoted the accumulation of flavonoids, anthocyanins and phenolics, and enhanced the antioxidant capacity of plants (Wang et al., 2020a; Zhang et al., 2025b). Additionally, blue light modulates the activity of DXS and DXR rate-limiting enzymes by regulating MEP pathway, influencing terpenoid biosynthesis (Lopes et al., 2020; Xie et al., 2021; Cheng et al., 2023). Molecular studies reveal that blue light stabilizes COP1 protein, altering the expression patterns of secondary metabolism-related genes (Liu et al., 2011; Wang et al., 2017; Miao et al., 2022). Optimizing blue light exposure can thus enhance bioactive compound production in medicinal plants and improve the nutritional quality of fruits and vegetables. Empirical studies demonstrate that blue light increases total flavonoids and phenolics in *Ocimum basilicum* (Fayezizadeh et al., 2024a), *Artemisia argyi* (Su et al., 2024), *Capsicum annuum* (Darko et al., 2022), *Rhodiola imbricata* (Kapoor et al., 2018), and *Brassica oleracea* (Lee et al., 2023), alongside elevated antioxidant activity. It also promotes JA (Zhang et al., 2023), terpenoid (Morello et al., 2022; Le et al., 2023), and alkaloid (Moranska et al., 2023) accumulation. In *Oryza sativa*, blue light upregulates PAL, 4CL, CHS, CHI, F3H, and FLS genes, enhancing enzyme activity and flavonoid biosynthesis (Zhang et al., 2025b).

In addition, blue light can interact with selenium nanoparticles(SeNPs) through specific photoreceptors, and the interaction between nano-materials and light environment can be used to regulate plant physiological and biochemical processes. For instance, Mazhar et al. (2024) treated *Santalum album* with SeNPs under blue LED light, significantly increasing total phenolics, saponins, terpenoids, and flavan-3-ols. In modern controlled-environment agriculture, blue LED lighting is widely adopted for tissue culture, leafy vegetable quality enhancement, and medicinal plant cultivation. However, optimal spectral parameters must be tailored to crop species and growth stages to maximize efficacy while avoiding photoinhibition.

### 2.3 Red light

**Figure 4:**
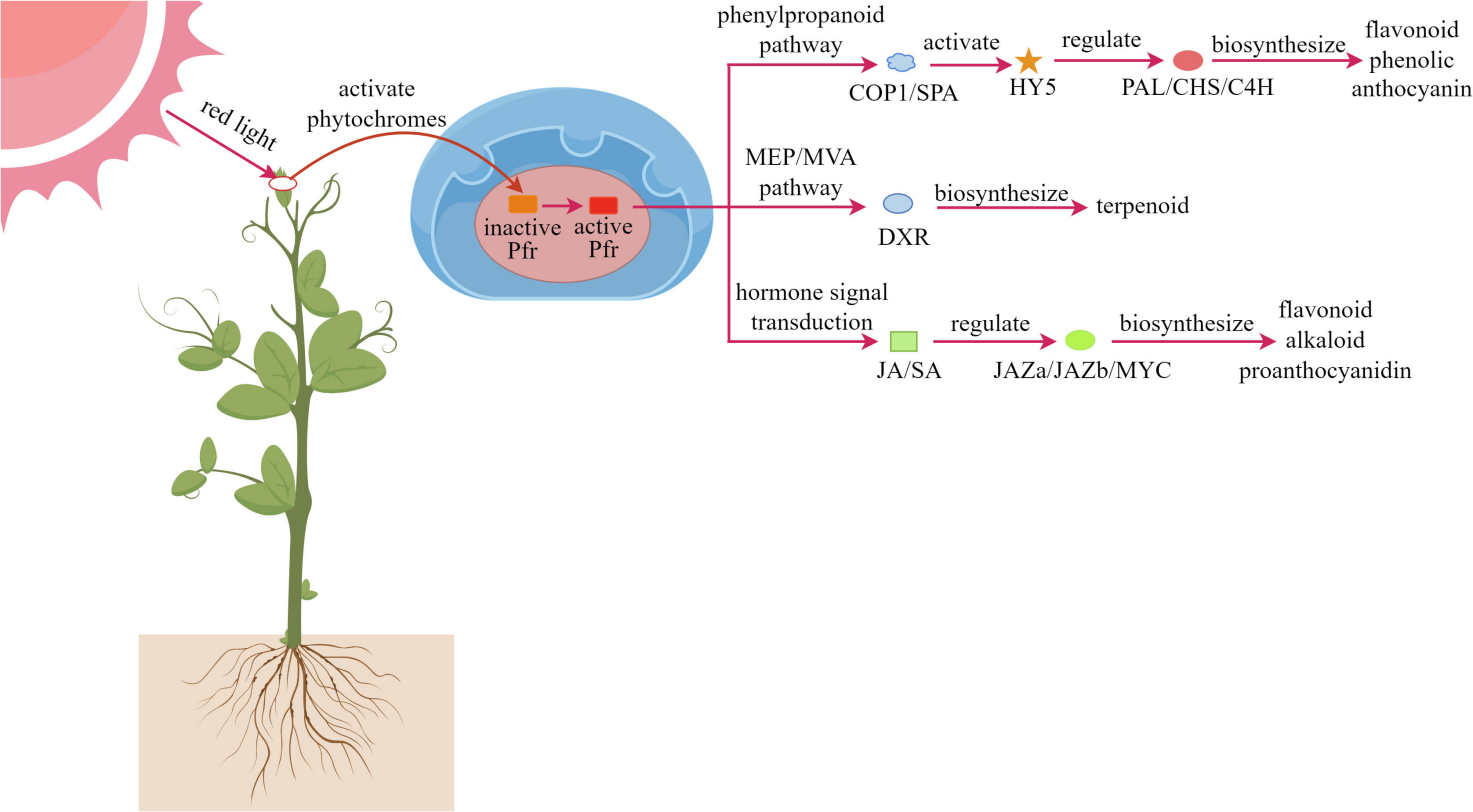
Mechanism of red light regulating plant secondary metabolites.

Phytochrome, as a light-sensitive protein complex, is widely distributed in plant cells. Red light can regulate physiological processes in plants through the phytochrome system, triggering a series of complex signal transduction pathways (De Wit et al., 2016; Holalu et al., 2020). As shown in **Figure 4**, When plants are exposed to red light, photoreceptors such as phytochromes are activated, and these signals are transmitted to the nucleus, triggering a cascade of molecular responses (Viczián et al., 2017; Kwon et al., 2024; Péter et al., 2024). First, the activated Pfr form can interfere with the function of the COP1/SPA protein complex, thereby stabilizing the expression of the HY5 transcription factor (Sweere et al., 2001; Oh et al., 2020). Second, red light upregulates PAL and CHS key enzyme genes in the phenylpropanoid metabolic pathway, promoting the synthesis of antioxidant compounds such as anthocyanins, flavonoids, and phenolics (Pashkovskiy et al., 2024; Zhang et al., 2024a). Within the MEP/MVA metabolic pathways, red light enhances the expression of the DXR gene, increasing terpenoid production (Lopes et al., 2020; Sankhuan et al., 2022b; Mokhtari et al., 2025). Additionally, red light signaling can synergize with plant hormone systems, particularly the JA and SA pathways, to coordinately regulate the biosynthesis of various defensive secondary metabolites, including anthocyanins and alkaloids (Li et al., 2014; Zhang et al., 2023).

These physiological changes collectively enhance the plant’s ability to cope with environmental stress. Pashkovskiy et al. (2023) demonstrated that under red and far-red light, the content of proanthocyanidins and catechins in *Scots Pine* are increased and the possible mechanism is to promote the expression of CHS gene. In *Triticum aestivum* treated with red light for four days, the activity and relative gene expression levels of PAL, C4H, and 4CL were upregulated, effectively stimulating the synthesis of total phenolics and enhancing antioxidant capacity (Zhang et al., 2024a). Under red light, polyphenol accumulation in *sage shoot* was promoted, accompanied by upregulated PAL, TAT, and RAS gene expression, leading to increased rosmarinic acid content (Grzegorczyk-Karolak et al., 2025). Red light also stimulated the accumulation of catechins, flavonoids, and proanthocyanidins in *Scots Pine*, likely by promoting the expression of 4CL and LAR genes, as well as the biosynthesis of PR1 and PR5 genes in the SA pathway and the relative expression of JA biosynthesis JAZa, JAZb, and MYC genes (Pashkovskiy et al., 2023). The putrescine biosynthesis pathway is an important precursor for the synthesis of tropane alkaloids, while red light enhances nitrogen metabolism and precursor synthesis in *Atropa belladonna* by regulating the expression of GDHA, At2g42690, and PAO5 genes, thus promoting the accumulation of tropane alkaloids (Gu et al., 2025). Therefore, red light can specifically activate plant photoreceptors and downstream metabolic pathways, directionally increase the content of plant secondary metabolites, and provide sustainable light regulation strategies for medicinal plant cultivation and agricultural development.

### 2.4 Green light

Green light, as an essential component of the visible spectrum, plays significant regulatory roles in plant physiology despite its low absorption efficiency by chlorophyll and other photosynthetic pigments. Through cryptochrome-mediated signaling pathways and other photoreceptors, green light not only influences photomorphogenesis and developmental processes but also modulates the biosynthesis and accumulation of secondary metabolites, serving crucial functions in environmental adaptation and defense mechanisms. At the molecular level, green light alters metabolic pathway activity by regulating the expression of PAL and CHS enzyme genes, thereby promoting or suppressing specific secondary metabolite production (Liu et al., 2018). Research demonstrates that green light at appropriate intensities can mitigate photo-oxidative damage while enhancing volatile compound accumulation (Lopes et al., 2020). Notably, green light counteracts blue light-induced suppression of HvNCED gene expression, thereby stimulating secondary metabolic processes (Hoang et al., 2014). Mechanistic studies reveal that green light promotes the accumulation of procyanidin B2/B3 and L-ascorbic acid in *Camellia sinensis* through downregulation of CRY2/3 and PHOT2 expression (Zheng et al., 2019). Furthermore, synergistic effects emerge when green light is combined with UV irradiation, significantly inducing the accumulation of phenolic compounds and phytohormones (Palma et al., 2022). These findings provide novel insights for light-quality regulation in controlled environment agriculture, particularly for enhancing medicinal compound production through optimized spectral combinations. Future research should focus on elucidating the interaction mechanisms between green light signaling and metabolic networks to facilitate its application in precision agriculture.

### 2.5 Multiple light qualities

Combined light quality application refers to the use of light with different wavelengths in specific ratios to collectively influence plant growth and development. In terms of the mechanism, different light qualities are perceived by plants through their specific photoreceptors, activating downstream signal transduction networks. This composite light environment, through synergistic regulation, can significantly affect the synthesis and accumulation of plant secondary metabolites, often yielding better results than single-light treatments. For example, in *Stevia rebaudiana*, the synthesis of phenolic compounds such as neochlorogenic acid, chlorogenic acid, and caffeic acid was significantly enhanced under two light combinations: 50% UV + 35% red + 15% blue and 50% far-red + 35% red + 15% blue (Ptak et al., 2024). After 24 days of red and blue light exposure, the levels of total anthocyanins, flavonoids, and phenolics increased in *Brassica rapa* (Bungala et al., 2024). Similarly, when *Cannabis sativa* was exposed to a light combination of 90% red, 8% blue, and 1% far-red for 43 days, the contents of 9-tetrahydrocannabinol and cannabidiol were increased (Carranza-Ramírez et al., 2025). Palma et al. (2022) showed that when *Cucumis sativus* was grown under green light for 9 days followed by UV supplementation for an additional 14 days, phenolic compounds increased, however, plants exposed to UV under a blue light background exhibited reduced metabolites associated with the hydrocinnamate or flavonoid pathway. Under mixed red-blue light (70% red + 30% blue), the accumulation of total phenolics and rosmarinic acid in *Melissa officinalis* was increased, which may be due to the up-regulation of genes related to primary metabolism (e.g., DAHPS for aromatic amino acids) and secondary metabolism (e.g., TAT, RAS for phenylpropanoid biosynthesis) (Ahmadi et al., 2022). Therefore, light quality combination technology enables targeted improvement of crop quality, offering new strategies for developing functional plant products and natural medicines. This multi-light synergistic regulation not only enhances the yield of secondary metabolites but also precisely modifies their compositional ratios, demonstrating broad prospects for future applications.

## 3 Effects of light intensity on plant secondary metabolites

**Figure 5:**
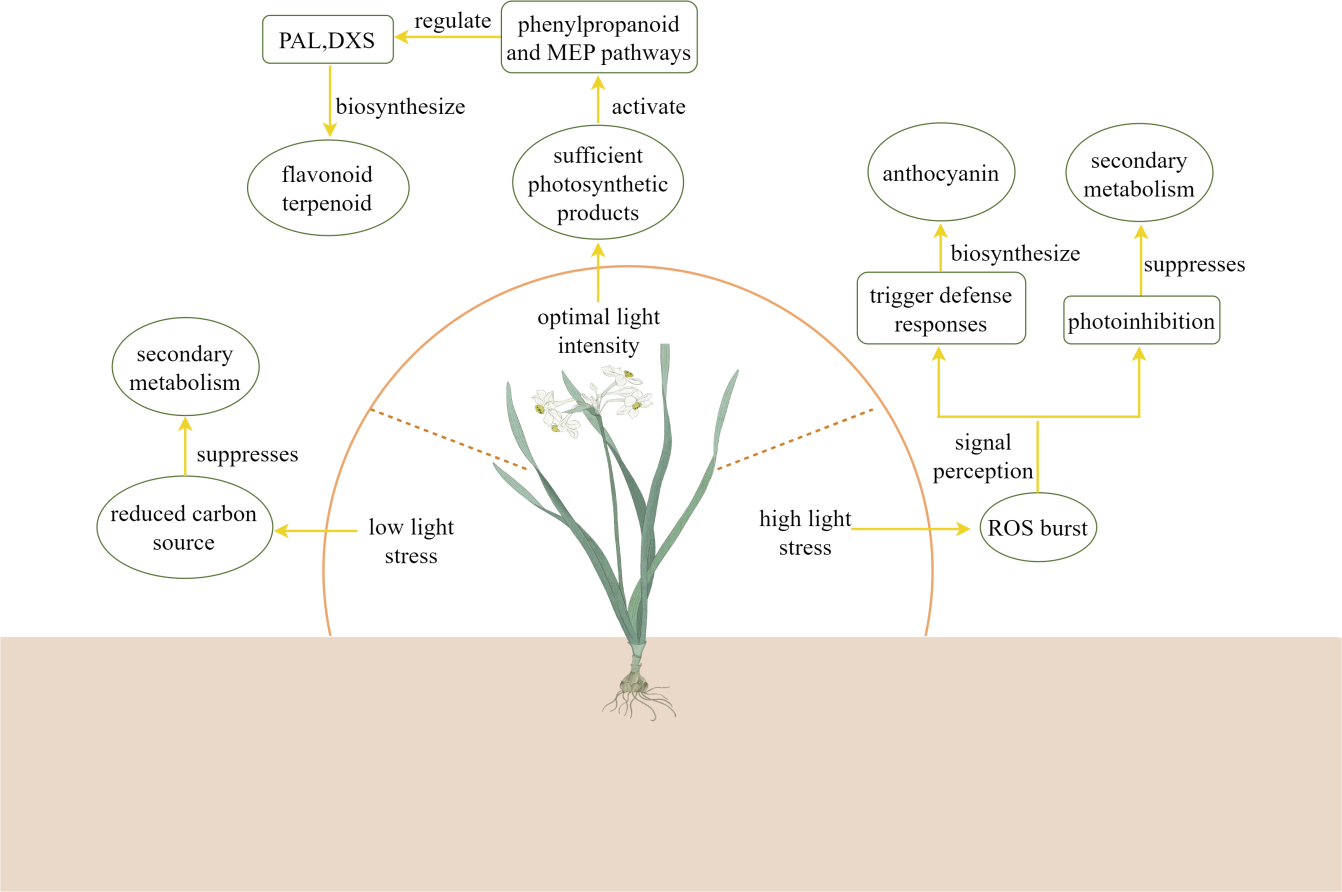
Effects of light intensity on the accumulation of plant secondary metabolites.

Light intensity is one of the key environmental factors influencing the biosynthesis of plant secondary metabolites. As shown in **Table 2**, the effects of different light intensity treatments on the secondary metabolites of plants varied. While different light intensities regulate the production and accumulation of various secondary metabolites by altering plant physiological states and metabolic pathways (Formisano et al., 2022). As illustrated in the **Figure 5**, under low light stress, carbon source accumulation in plants is suppressed, thereby inhibiting secondary metabolite biosynthesis (Shi et al., 2015). Within optimal light ranges, plants achieve peak photosynthetic efficiency, providing sufficient carbon sources and energy to support secondary metabolism (Wang et al., 2020b; An et al., 2022). Under these conditions, plants activate multiple metabolic pathways: they enhance phenylpropanoid metabolism to increase synthesis of antioxidant compounds like flavonoids and phenolics (Liu et al., 2019), while simultaneously stimulating production of terpenoids including volatile monoterpenes and sesquiterpenes (Reichel et al., 2022). However, under high-light stress, the burst of ROS in plants damages the photosynthetic apparatus, reducing ATP/NADPH supply (Borbély et al., 2022). In response to stress signals, plants initiate protective responses (Zhang et al., 2025a). At this time, photosystem II activates defense mechanisms through signal transduction pathways that induce synthesis of protective compounds (Wu et al., 2016). This process leads to significant accumulation of photoprotective pigments such as anthocyanins and carotenoids, along with increased production of defensive secondary metabolites like alkaloids (Zhang et al., 2019). Alternatively, when photoprotection fails, photoinhibition occurs, negatively impacting secondary metabolite biosynthesis (Huang et al., 2022a). Studies demonstrate that high-intensity light stress induces photo-oxidation in etiolated *Camellia sinensis*, which upregulates the expression of CHI and F3’H genes through the dihydroxy flavonoids and xanthophyll cycle pathways. This metabolic response enhances the accumulation of flavonoid antioxidants such as quercetin, thereby establishing a photoprotective mechanism via ROS scavenging to mitigate light stress tolerance (Zhang et al., 2017). Cao et al. (2024) compared different light intensities in *Anoectochilus roxburghii* cultivation and measured anthocyanin content. The results demonstrated that under 75% transmittance (80 μmol·m^2^·s^1^), the expression levels of CHS, FLS, and F3’H genes were significantly upregulated, leading to increased anthocyanin accumulation. High-intensity light irradiation promotes both a higher net photosynthetic rate and upregulation of PAL, 4CL, and C4H enzyme expression in *Asarum heterotropoides*, consequently enhancing essential oil biosynthesis (Wang et al., 2020b). Under high light intensity (400 μmol·m^2^·s^1^), the gene expression of CsCHIa, CsCHIb, CsF3’Hb, CsF3’Hc, CsDFRb2, and CsLARd in *Camellia sinensis* was downregulated, leading to reduced accumulation of naringenin, dihydroquercetin, leucocyanidin, and catechin (Zhang et al., 2025a). As a key gene of nicotine biosynthesis in *Nicotiana attenuata*, NaABI4 activates the gene expression of NaJAT1/2 and NaHY5 under the light intensity of 300 μmol·m^-2^·s^-1^, increases the accumulation of nicotine and promotes nicotine biosynthesis (Lei et al., 2025).

**Table 2:** Effect of light intensity on accumulation of secondary metabolites in plants.

Species	Light environment	Regulated genes	Secondary metabolites	References
*Nicotiana attenuata*	300 μmol·m^-2^·s^-1^	up-regulating NaJAT1/2, NaHY5	nicotine↑	(Lei et al., 2025)
*Bletilla striata*	50% shading for 60 days	down-regulating C4H, HCT, CSE, F5H, CHS, DXS	cinnamic acid, chlorogenic acid, naringenin, daidzein, carvone and gossypol ↓	(Xu et al., 2024)
*Syringa oblata*	40% ~ 50% shading for 4 months	up-regulating 4CL1, CYP73A, CYP75B1	rutin and flavonoids↑	(Liu et al., 2019)
*Origanum majorana*	30% shading for 60 days	up-regulating DXR, CYP771D179, CYP71D178	essential oil↑	(Hashemifar et al., 2024)
*Catharanthus roseus*	80 μmol·m^-2^·s^-1^ for 23 days	up-regulating LAR	fraxin, quercetin↓	(Gholizadeh et al., 2023)
*Angelica dahurica*	90% shading for 60 days	down-regulating 4CL, COTM	coumarin↓	(Huang et al., 2022b)
*Anoectochilus roxburghii*	80 μmol·m^-2^·s^-1^ for 15 days	up-regulating CHS, FLS, F3′H	anthocyanin↑	(Cao et al., 2024)
*Vitis vinifera*	150 μmol·m^-2^·s^-1^	up-regulating VvFLS1	flavonol↑	(Downey et al., 2003)
*Camellia sinensis*	400 μmol·m^-2^·s^-1^ for 14 days	down-regulating CsCHIa, CsCHIb, CsF30 Hb, CsF30Hc, CsDFRb2, CsLARd	naringenin, dihydroquercetin, leucocyanidin, and catechin ↓	(Zhang et al., 2025a)

↑ represents increase, ↓ represents decrease.

Conversely, under low light stress, plants prioritize energy allocation to essential metabolic processes, often reducing synthesis of complex secondary metabolites while maintaining certain specific compounds at elevated levels (Huang et al., 2022b; Kamal et al., 2022). Yamawo and Tomlinson (2023) showed that the total phenolic content in *Aralia elata* decreased under low light conditions. In *Bletilla striata*, 50% shading (transmitting 50% natural sunlight) for 60 days downregulates genes involved in phenylpropanoidand and flavonoid(C4H, HCT, CSE, F5H, CHS) biosynthesis, as well as the terpenoid pathway gene DXS, reducing corresponding metabolite levels (Xu et al., 2024). *Mangifera indica* bagging treatments upregulate LAR expression while suppressing proanthocyanidin accumulation in fruit peel, with additional modulation of flavonoid biosynthesis through MYB, bHLH, ERF, WRKY and bZIP gene regulation (Qian et al., 2023). In *Angelica dahurica*, Huang et al. (2022b) observed that 90% shading downregulates 4CL and COTM to inhibit coumarin biosynthesis, while 50% shading upregulates PAL to promote coumarin accumulation. Compared to full sunlight (0% shading), 30% shading increased the content of total phenolics, total flavonoids, and chlorogenic acid in *Ipomoea batatas* (Jing et al., 2023). As a shade-tolerant plant, *Glechoma longituba* exhibits increased aboveground dry matter yield and enhanced accumulation of ursolic acid and oleanolic acid under appropriate shading, thereby improving its medicinal value (Zhang et al., 2015). Compared to normal light intensity (1000 μmol·m^2^·s^1^), low-light stress (250 μmol·m^2^·s^1^) suppressed carotenoid and anthocyanin biosynthesis in *Brassica campestris*, resulting in a phenotypic shift from purple to green leaves (Zhu et al., 2017). Therefore, these findings demonstrate that precise light intensity regulation represents a powerful tool for targeted enrichment of valuable metabolites in medicinal plant cultivation.

## 4 Effects of photoperiod on plant secondary metabolites

Photoperiod is a crucial environmental signal regulating plant physiological metabolism, which modulates the biosynthesis dynamics of plant secondary metabolites through the circadian clock system and light signal transduction network. The primary mechanism involves plants perceiving changes in day length via specific photoreceptors (e.g., phytochromes, cryptochromes) and transmitting light signals to the central circadian clock, ultimately forming a cascade response system of light signal-circadian rhythm-metabolic network (Flis et al., 2016; Djerrab et al., 2021; Liu et al., 2023; Pérez-Llorca and Müller, 2024). At the metabolic regulation level, the photoperiod effect is mainly reflected in regulating the diurnal expression patterns of key enzymes PAL and TAL in the phenylpropanoid pathway, influencing the synthesis rhythm of flavonoids and phenolic compounds (Vazirifar et al., 2021; Fukuda et al., 2022). Additionally, photoperiod affects the periodic accumulation of terpenoids (Ali et al., 2018; D’aquino et al., 2024) and the synthesis dynamics of nitrogen-containing secondary metabolites (Campos-Tamayo et al., 2008; Akhgari et al., 2015), which may be involved in the plant’s adaptation to light environments. From an ecological perspective, photoperiod-regulated metabolite changes reflect plant adaptation strategies to seasonal environments. As shown in **Table 3**, photoperiodic changes regulate the accumulation of plant secondary metabolites.

**Table 3:** Effect of photoperiod on accumulation of secondary metabolites in plants.

Species	Class	Light condition	Metabolites	References
*Litsea cubeba*	terpenoid	darkness treatment 18 h day^-1^	monoterpenes ↓	(Yang et al., 2025)
*Ocimum basilicum*	polyphenols, flavonoid	continuous light 24 h day^-1^	total phenolic, total flavonoid, anthocyanin ↑	(Fayezizadeh et al., 2024b)
*Petunia hybrida*	flavonoid	12h/12h light/dark cycle for 14 days	anthocyanin ↑	(Pérez-García et al., 2015)
*Caralluma tuberculata*	phenolic, flavonoid	darkness treatment for 14 days	coumarins, gallic acid, caffeic acid, ferulic acid, catechine, quercetin, rutin ↑	(Ali et al., 2023)
*Moringa oleifera*	phenolic, flavonoid	continuous light 24 h day^-1^ for 28 days	kaempferol, neochlorogenic acid, quercetin ↑	(Bajwa et al., 2023)

↑ represents increase, ↓ represents decrease.

Long-day conditions promote the synthesis of pigments and volatile compounds related to reproduction. In *Litsea cubeba*, dark treatment significantly reduces monoterpene content in fruits and suppresses gene expression in the MVA and MEP pathways (Yang et al., 2025). Studies have shown that basil exhibits increased levels of total phenolics, flavonoids, and anthocyanins under a 24-hour photoperiod (Fayezizadeh et al., 2024b). Continuous white light (24 hours) for 28 days increases total phenolics and flavonoids (e.g., kaempferol, neochlorogenic acid, quercetin) in *Moringa oleifera* (Bajwa et al., 2023). Moreover, the accumulation of anthocyanins, carotenoids and flavonoids in plants is regulated by the circadian clock and adapts to photoperiod changes, thereby maintaining circadian clock integrity (Covington et al., 2008; Hildreth et al., 2022; Odgerel et al., 2022). When the photoperiod changes, the regulation of circadian rhythm-related processes affects phenylpropanoid biosynthesis in *Populus tremula* (Hoffman et al., 2010). In *Arabidopsis thaliana*, light regulates the interaction between the circadian transcription factors RVE8 and LNK, thereby promoting anthocyanin accumulation (Pérez-García et al., 2015). Studies demonstrate that extended photoperiods enhance the accumulation of volatile compounds in *Ocimum basilicum*, including linalool, eucalyptol, and eugenol (D’aquino et al., 2024). Many genes in the phenylpropanoid biosynthesis pathway are controlled by the circadian clock and phenylpropanoid-derived secondary metabolites in *Arabidopsis thaliana* are synthesized to protect cells from photoinhibition (Harmer et al., 2000). In *Arabidopsis thaliana*, the expression of AtMYB genes is regulated by the circadian clock and promotes anthocyanin biosynthesis in a circadian clock-dependent manner (Nguyen and Lee, 2016). Light promotes glucosinolate biosynthesis in *Brassica rapa* by modulating the circadian clock gene BrGI (Kim et al., 2021). However, short-day conditions induce the accumulation of stress-resistant metabolites. Ali et al. (2023) cultivated *Caralluma tuberculata* in complete darkness for 2 weeks with 100 μg/L SeNPs, then transferring them to normal light until day 56, which showed increased contents of coumarin, gallic acid, caffeic acid, ferulic acid, catechin, quercetin, and rutin. Supplementing *Solanum lycopersicum* with 3-hour morning light boosts phenolics accumulation but reduces flavonoids, whereas 3-hour evening light increases flavonoids without significantly affecting phenolics (Wang et al., 2022). The studies showed that *Begonia semperflorens* seedlings grown under short days accumulate more carbohydrates and ABA, both of which induce anthocyanin biosynthesis (Zhang et al., 2016). Thus, the core of these “time-dependent metabolic regulation” mechanisms lies in the circadian clock system’s integration and transduction of environmental signals, precisely controlling secondary metabolic pathway activity to optimize plant ecological adaptability (Seaton et al., 2018). This principle holds significant application value in medicinal plant cultivation and specialty agricultural production, providing a theoretical foundation and technical pathway for the temporal precision regulation of metabolites.

## 5 Light-induced secondary metabolism in plant organs and its technical applications

Light exerts crucial effects on plant growth and development, not only by providing energy for photosynthesis but also by activating multiple signaling pathways to regulate secondary metabolism. Plants perceive light signals through photoreceptors such as phytochromes and cryptochromes, which collaborate with transcription factors (e.g., HY5, PIFs) to form a sophisticated light-signaling network, modulating the synthesis of metabolites in different tissues. Due to structural and functional differentiation among plant organs, leaves, flowers, fruits, and roots exhibit distinct responses to light environments, which determines the distribution patterns of various metabolites. Upon light signal perception by photoreceptors, plants initiate tissue-specific response programs that regulate secondary metabolism through hormonal signaling and transcriptional networks. Taking leaves as an example, their photosynthetic and defensive functions are closely light-dependent. Blue or far-red light activates the leaf photoreceptor system, which modulates MYB transcription factors to alter the phenylpropanoid pathway, thereby enhancing antioxidant production (Zhou et al., 2024). Different light radiation triggers intracellular signaling through specific receptor systems, thereby regulating the synthesis of secondary metabolites such as phenylpropanoids and terpenoids. This process determines the accumulation patterns of defense-related compounds, pigments, or pharmaceutically valuable substances in leaves (Zhu et al., 2017). As crucial reproductive structures in plants, flowers not only facilitate pollination but also serve as biosynthesis sites for diverse secondary metabolites, with their development and metabolic activities being light-regulated. The photoreceptor systems distributed in flowers perceive and transduce light signals, subsequently activating transcription factors (e.g., HY5, MYBs) to modulate phenylpropanoid and terpenoid metabolic pathways (Marzi et al., 2020). Physiologically, light influences the synthesis of anthocyanins and other pigments that determine floral coloration, while simultaneously regulating the accumulation of volatile aromatic compounds and defensive metabolites (Guo et al., 2018; Li et al., 2020). These light-mediated changes carry significant ecological implications: floral pigmentation and fragrance attract pollinators, while defensive compounds enhance reproductive success, thereby optimizing the coordination between developmental processes and metabolic homeostasis. As an essential component of plant reproductive systems, fruits undergo light-regulated physiological processes affecting pigmentation and ripening (Dzakovich et al., 2016). In *Solanum lycopersicum*, UV-B radiation significantly enhances carotenoid and anthocyanin biosynthesis through the HY5-mediated signaling pathway (Abramova et al., 2023). Furthermore, under 150 µmol·m^2^·s^1^ light intensity, upregulated expression of the VvFLS1 gene in *Vitis vinifera* promotes flavonol production (Downey et al., 2003). Although plant roots do not directly perform photosynthesis, their growth and metabolism remain under photoregulation. This regulation primarily involves light signal perception and response - root tips detect red light signals through phytochromes to trigger negative phototropism, forming characteristic skotomorphogenic growth patterns as demonstrated by inhibited root elongation in *Arabidopsis thaliana* (Van Gelderen et al., 2018; Spaninks and Offringa, 2023). Furthermore, shoot-to-root communication influences root systems through photosynthetic assimilate allocation, phytohormone network regulation, and systemic signaling transduction three pathways (Valbuena et al., 2018; Leschevin et al., 2024).

The application of light-regulated plant secondary metabolites refers to the targeted induction of functionally specific secondary metabolite biosynthesis in plants through manipulation of light parameters, enabling their industrial utilization in agriculture, medicine, food production, and other fields. In enclosed cultivation systems such as photobioreactors and artificial climate chambers, spectral-tunable LED panels are used to optimize metabolite production by selecting specific wavelength combinations for different metabolites, employing photosensors for real-time monitoring and automatic adjustment of light intensity, and designing photoperiods according to plant circadian rhythms, thereby maximizing the accumulation of secondary metabolites (Hoffman et al., 2010). In vertical farming systems, a stratified lighting strategy with three-dimensional spectral recipes can be implemented: upper layers utilize UV-B to enhance flavonoid production, middle layers employ blue-red light combinations to increase fresh and dry weight, while lower layers apply far-red light to stimulate the accumulation of root bioactive compounds (Larsen et al., 2020; Yoon et al., 2021; Kim et al., 2025). Greenhouse control systems can integrate natural sunlight with LED supplemental lighting to automatically activate specific light wavelengths during low-light seasons, ensuring stable metabolite production. Additionally, stress-induction techniques can be employed, such as short-term high-intensity illumination or UV pulsed irradiation, to activate plant defense metabolic pathways. During the postharvest storage of plants, light treatment technology can effectively preserve and enhance functional components. For example, blue light irradiation during *Solanum lycopersicum* storage helps delay softening, reduce decay, and better maintain fruit quality (Grzegorzewska et al., 2024). Brief UV-C irradiation applied to postharvest *Vigna radiata* effectively reduces fresh weight loss, while increasing total phenolics, protein content, and antioxidant capacity (Tripathi et al., 2024). Under 10°C storage conditions, red-blue LED treatment extends the shelf life of *Brassica oleracea* by 4 days (Liu et al., 2025). Based on these findings, an integrated LED lighting and temperature-controlled storage system was developed to achieve superior plant preservation, enhanced quality, and maintained bioactivity.

## 6 Discussion

### 6.1 Light signaling regulates the production of plant secondary metabolites

Light environment is a key ecological factor influencing plant physiological processes, and its regulatory role in secondary metabolites has become a research hotspot in the field of plant science. Studies have shown that light quality, intensity, and photoperiod can regulate the biosynthesis and accumulation of plant secondary metabolites through complex photoreceptor systems, such as phytochromes, cryptochromes, and UVR8 receptor proteins, and signal transduction networks (Ballaré, 2014; Taulavuori et al., 2017; Tossi et al., 2019). This light-mediated regulation not only leads to quantitative changes in end-product content but also affects the expression of key enzyme genes in metabolic pathways (Hideg et al., 2013; Li et al., 2017). In-depth exploration of this regulatory mechanism holds significant value for understanding the evolutionary history of plant photoadaptation and developing light-based strategies for crop quality improvement. From a fundamental research perspective, it provides new insights into the molecular mechanisms of plant environmental adaptation and the regulatory principles of metabolic networks. In practical applications, precise control of light environment parameters can effectively optimize the active compound profiles of medicinal plants and enhance the nutritional and functional properties of agricultural products, which lays a technological foundation for modern facility agriculture and industrialized plant production systems. To leverage the potential of light-regulated plant secondary metabolite accumulation, we can utilize precisely tuned light wavelengths and intensities to achieve targeted biosynthesis of desired metabolites. This integrated approach combines plant photobiology, metabolic pathway characteristics, and engineered control methods, featuring dynamic multi-wavelength switching through tunable LED arrays optimized for different metabolic stages, light intensity gradient optimization with photosensor-enabled real-time adjustment to prevent photoinhibition, and temporal light modulation synchronized with circadian rhythms for enhanced pathway efficiency. Currently, LED-based spectral modulation technology has been widely applied in the commercial cultivation of various medicinal crops, significantly improving the production efficiency of target bioactive compounds (Sankhuan et al., 2022a; Dsouza et al., 2024). These light-regulation technologies exhibit broad application potential, including the production of high-value therapeutics like paclitaxel in optically controlled bioreactor systems (Fernie et al., 2024). LED lighting technology enhances the nutritional quality of *Ocimum basilicum* and *Mentha canadensis* plants (Jakubczyk et al., 2024). Developing photo-optimized stress-resistant crops to address climate change challenges in agricultural production (Zhao et al., 2023). Furthermore, light environment regulation has been successfully employed in the targeted cultivation of specialty functional crops, such as glucosinolate-rich *Broccoli oleracea* (Wang et al., 2021) and high-flavonoid-content *Eruca sativa* (Veremeichik et al., 2023). Future research must achieve breakthroughs in key technologies including intelligent light-control systems and dynamic metabolic modeling, while evaluating the adaptability between laboratory photic conditions and natural environments, along with potential ecological risks.

### 6.2 Integration of light signal transduction with metabolic and genetic regulatory networks

Elucidating the integration mechanism between the light signaling system and the plant secondary metabolic network is a core scientific question that urgently needs to be addressed in current research. Although regulatory factors such as HY5 and MYB are involved in light-responsive metabolic processes, the synergistic mechanisms among these core transcriptional regulators and their dynamic response patterns to different light environments still require in-depth exploration (Jiang et al., 2012; Job and Datta, 2021; Chang et al., 2025). In particular, there remain significant research gaps in understanding how the interaction between light signals and abiotic stress factors such as temperature, water, and salinity regulates secondary metabolic pathways. Another key scientific question worthy of further investigation is the species-specific differences in light regulation observed among different plant groups. Studies have shown that due to variations in evolutionary background and ecological adaptation strategies, different plant species may exhibit markedly distinct metabolic responses to the same light conditions (Siatkowska et al., 2021; Jakubczyk et al., 2024). Taking light-adapted plants as an example, sun-loving and shade-tolerant plants may have fundamental differences in the molecular mechanisms of light regulated secondary metabolism, posing challenges for developing precise light regulation strategies in crops (Zhang et al., 2017; Tavridou et al., 2020). From a technological application perspective, integrating light environment control with metabolic engineering is regarded an innovative strategy to improve the production of target metabolites. In addition, by modifying light responsive elements or optimizing key transcriptional regulators, it may be possible to achieve precise regulation of plant secondary metabolic pathways (Riaz et al., 2019; Shi et al., 2024). Current advances in genetic and metabolic engineering focus on wavelength-specific photosensitive promoters (e.g., UV-responsive synthetic elements) to enable spatiotemporal control of metabolic pathways. AI-assisted photoreceptor engineering enhances environmental adaptability, while integrated ROS-sensing systems coordinate antioxidant defenses with secondary metabolite production. Key challenges include:incorporation of extremophile-derived resistance genes for improved stress tolerance, optimization of non-photochemical quenching mechanisms, and dynamic balancing between stress resilience and metabolic yield efficiency. However, such modifications may interfere with normal plant physiological and developmental processes, so it is necessary to find a balance between metabolic regulation and plant growth.

### 6.3 Intelligent regulation of light environment and multi-omics integrated research

Building an intelligent light environment regulation system may become a key research direction in the future. By leveraging artificial intelligence technology, a real-time monitoring network for plant physiological indicators and environmental parameters can be established to dynamically optimize light parameters and achieve precise regulation of secondary metabolites. This technological framework holds broad application prospects in plant factories and medicinal plant cultivation (Luna-Maldonado et al., 2016; Ren et al., 2023). Additionally, understanding the synergistic regulation mechanisms of multiple environmental factors is of significant research value. Investigating the interactions between light and key environmental variables such as CO_2_ concentration, temperature, and humidity, and constructing a multi-dimensional environmental regulation model will provide theoretical support for developing new and efficient cultivation methods (Han et al., 2024; Tanigawa et al., 2024). Against the backdrop of increasingly severe global climate change, conducting such multi-factor coupling studies is particularly urgent, offering critical implications for agricultural practices in response to climate challenges.

With advancements in photobiology, metabolomics, and synthetic biology, research on light mediated regulation of plant secondary metabolism is in a rapid development phase, deepening our understanding of complex regulatory systems (Bobrovskikh et al., 2022; Liao et al., 2024; Bechtold et al., 2025). Looking forward to the future, there is an urgent need to integrate multi-omics technologies, including genomics, transcriptomics, proteomics, and metabolomics, to systematically elucidate the molecular mechanisms of light-regulated secondary metabolism and translate fundamental research into practical applications, so as to give full unlock the potential of light-regulation technology in agricultural upgrading, drug development, and ecological conservation. In conclusion, light precisely regulates plant secondary metabolism through wavelength specificity, intensity, and duration. The core of these regulatory systems lies in photoreceptor-mediated signal perception, which triggers cascade reactions that activate specific transcription factors, thereby modulating the expression and activity of key metabolic enzymes (Qian et al., 2020). Based on this principle, optimizing crop quality or medicinal components using artificial light sources provides an innovative solution for replacing traditional chemical inducers with optical fertilizers, and also opens up new possibilities for green agriculture and sustainable development.

## Glossary

UV: Ultraviolet light
HY5: hypocotyl 5
UVR8: UV Resistance Locus 8
MYB: myeloblastosis
COP1: constitutive photomorphogenic 1
CRY: cryptochrome
PHOT: phototropin
PHY: phytochrome
MEP: methylerythritol 4-phosphate
MVA: mevalonic acid
JA: jasmonic acid
SA: salicylic acid
ABA: abscisic acid
ROS: reactive oxygen species
LED: light emitting diodes
TL: Tubular Luminescent
FL: fluorescent lamp
CHS: chalcone synthase
PAL: phenylalanine ammonia-lyase
C4H: cinnamate 4-hydroxylase
4CL: 4-coumarate-CoA ligase
CHI: chalcone isomerase
F3’H: flavanone 3’-hydroxylase
FLS: flavonol synthase
GDHA: glutamate dehydrogenase A
PAO5: polyamine oxidase 5
JAZa: jasmonate ZIM-domain protein a
HSF: heat shock factor
C3H: Cys3/His
bHLH: basic Helix-Loop-Helix
CCR: cinnamoyl-CoA reductase
POD: peroxidase
ANR: anthocyanidin reductase
TAT: tyrosine aminotransferase
RAS: rosmarinic acid synthase
DHD: dihydrodipicolinate
SDH: shikimate dehydrogenase
LAR: leucoanthocyanidin reductase
PR1: pathogenesis-related protein 1
PR5: pathogenesis-related protein 5
JAZb: jasmonate ZIM-domain protein b
MYC: myelocytomatosis
COI1: coronatine-insensitive protein 1
JAR1: JA-amino synthetase
DAHPS: 3-deoxy-D-arabino-heptolusonate 7-phosphate synthase
DFR: dihydroflavonol 4-reductase
STS: stilbene synthase
ADS: amorpha-4,11-dienesynthase
HMGR: 3-hydroxy-3-methylglutaryl CoA reductase
DXS: 1-deoxy-D-xylulose-5-phosphate synthase
DXR: 1-deoxy-D-xylulose-5-phosphate reductoisomerase
GGPPS: geranylgeranyl diphosphate synthase
CPS: copalyl diphosphate synthase
HCT: hydroxycinnamoyl-CoA transferase
CSE: caffeoyl shikimate esterase
F5H: ferulate 5-hydroxylase
COMT: caffeic acid O-methyltransferase.
